# Means of Solidifying Foot Prints

**Published:** 1851-07

**Authors:** 


### CHEMISTRY.

*Means of Solidifying Foot Prints.*—It is often a matter of great importance in medico-legal investigations, to preserve an accurate mould of foot-prints; for this purpose M. Hugoulin (*Annales d' Hygiene*) recommends the following plan:—An iron plate supported by bars, is so placed as to be about three or four *centimetres* above the mark to be solidified, and upon it live coals are to be placed so that heat may radiate to the subjacent soil. When this has become heated to about 100°C., *stearic acid*, reduced to impalpable powder, (by previously dissolving it in its weight of alcohol, and then having added abundance of water, evaporating,) is to be dusted over it through a fine hair seive, so as to form an uniform layer. This, falling as a snowy dust, cannot by its weight injure the impression, and is indeed dissolved and absorbed as soon as it touches the soil. It is continued until the earth has become too cold to dissolve any more. After a sufficient time has been allowed for it to have become completely cold, the earth is mined round at some distance so as to raise the entire mass in a single piece, cutting afterwards carefully away the superfluous soil. After reversing it on several folds of linen, and surrounding it with a case, plaster is allowed to run in between the case and the reverse of the impression, so as to ensure complete solidity to it. If the soil is muddy or marshy, before commencing these operations, a trench should be dug around, into which plaster is to be introduced, which, on solidifying, will absorb much of the moisture, after which the whole mass may be exposed for some days to the sun and air before commencing to act upon it. M. Hugoulin says this plan is applicable to the most shifting soils, as sand &c., and indeed to all except snow.—*Prov. Med. and Surg. Jour.*

